# Supplementary figures and images for: Single Cell RNA Sequencing Reveals Critical Functions of Mkx in Periodontal Ligament Homeostasis
Source: Front Cell Dev Biol. 2022 Feb 4;10:795441. doi: 10.3389/fcell.2022.795441 (PMC8854991; doi:10.3389/fcell.2022.795441)

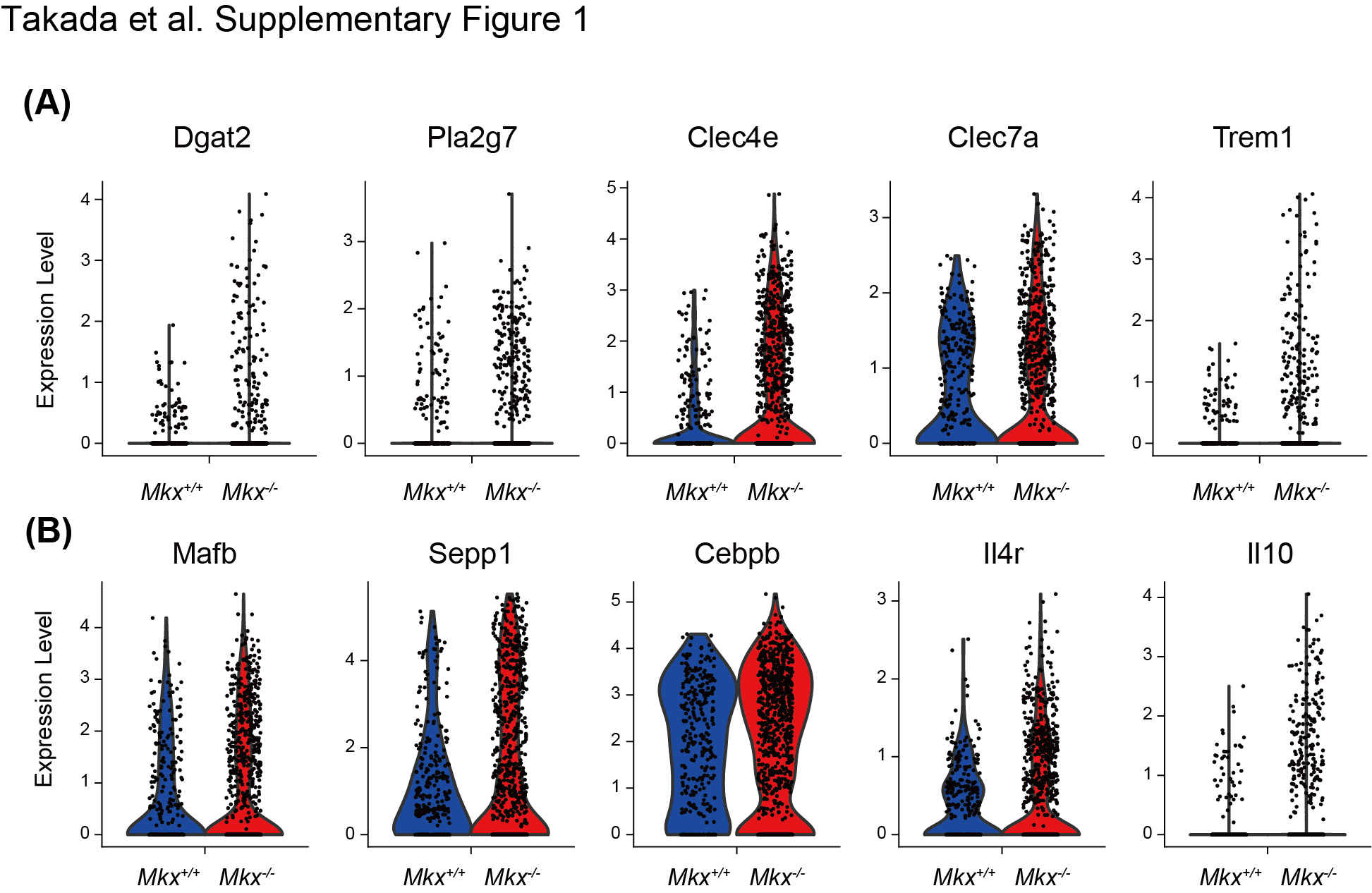

Supplement: Supplementary file 1 [file Image1.JPEG]

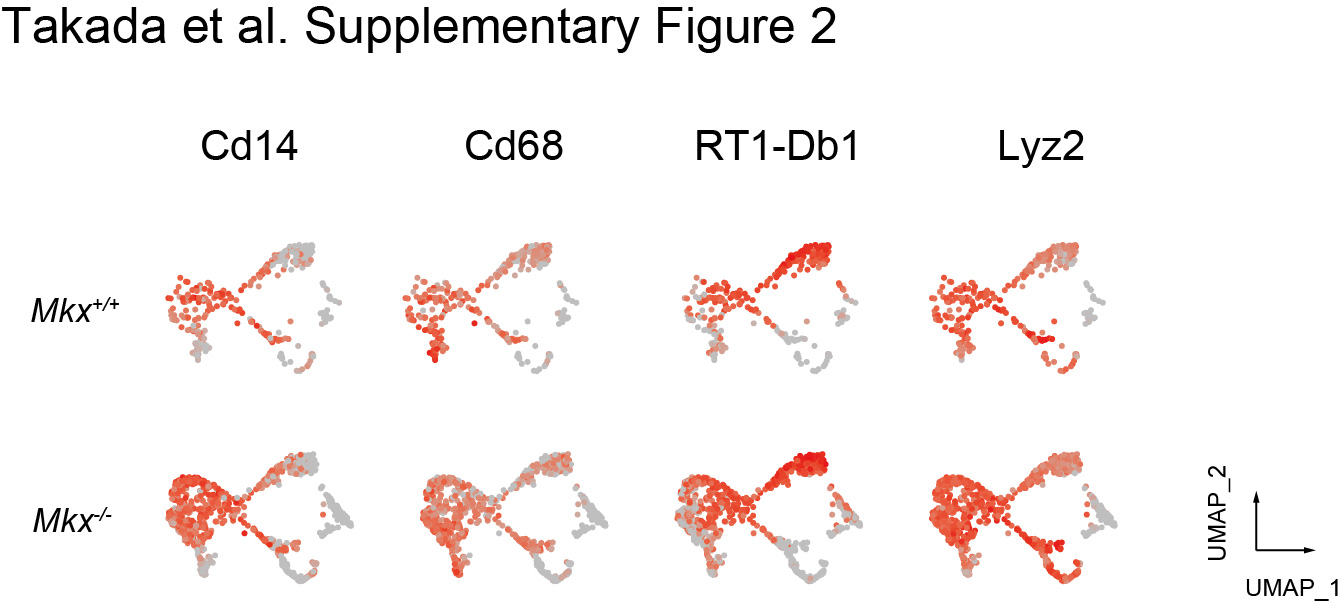

Supplement: Supplementary file 2 [file Image2.JPEG]
